# Diagnosis of patients with angina and non-obstructive coronary disease in the catheter laboratory

**DOI:** 10.1136/heartjnl-2019-315042

**Published:** 2019-07-31

**Authors:** Haseeb Rahman, David Corcoran, Muhammad Aetesam-ur-Rahman, Stephen P Hoole, Colin Berry, Divaka Perera

**Affiliations:** 1 The BHF Centre of Excellence and the NIHR Biomedical Research Centre at the School of Cardiovascular Medicine and Sciences, King’s College London, London, UK; 2 British Heart Foundation Glasgow Cardiovascular Research Centre, Institute of Cardiovascular and Medical Sciences, University of Glasgow, Glasgow, UK; 3 Department of Cardiology, Papworth Hospital NHS Foundation Trust, Cambridge, UK

**Keywords:** chronic coronary disease, cardiac catheterization and angiography, quality and outcomes of care

## Abstract

Around 40% of all patients undergoing angiography are found to have normal coronary arteries or non-obstructive coronary artery disease (NOCAD). Despite the high prevalence, this is a group who rarely receive a definitive diagnosis, are frequently labelled and managed inappropriately and by and large, continue to remain symptomatic. Half of this group will have coronary microvascular dysfunction (CMD), associated with a higher rate of major adverse cardiovascular events; identifying CMD represents a therapeutic target of unmet need. As the pressure wire has revolutionised our ability to interrogate epicardial coronary disease during the time of angiography, measuring flow can similarly classify NOCAD during a single procedure. Assessment of flow is a function that is already integral to some pressure wires and furthermore, the familiarity and usage of the combined Doppler and pressure wire is rapidly increasing—these are techniques that readily lend themselves to the skillset of a practising interventional cardiologist. We present a structured algorithm designed for cardiologists who frequently encounter NOCAD in the catheter laboratory, identifying specific disease phenotypes within this heterogeneous population with linked therapy. This review paper clearly explains the rationale for this algorithm and outlines its applicability to routine clinical practice and also, the importance of phenotyping for future research. Ultimately, personalised therapy could improve outcomes for both patients and healthcare providers; while these approaches in turn will need robust evaluation to ensure that they improve both clinical outcomes and health economic benefits, this proposal will provide a framework for future trials and evaluations.

## Clinical context

The standard of care for investigating stable angina in patients with an intermediate-high likelihood of coronary artery disease (CAD) is the coronary angiogram. In a registry of nearly 400 000 patients undergoing invasive coronary angiography, approximately 40% had Non-Obstructive Coronary Artery Disease, a diagnosis often referred to as ‘NOCAD’.^1^ NOCAD is associated with worse healthcare outcomes and higher economical costs than previously appreciated.^2^ This umbrella term encompasses a broad range of cardiac pathophysiological abnormalities, including endothelial dysfunction, microvascular remodelling (structural), microvascular and epicardial spasm (functional), vasomotor abnormalities and enhanced cardiac pain perception in addition to non-cardiac chest pain. Demonstrable ischemia with normal coronary arteries (INOCA) is a recent term used to describe subsets of NOCAD patients, however this neither distinguishes aetiology nor involves direct assessment of the microvasculature.^3^ In 1988, the term coronary microvascular dysfunction (CMD) was used to refine this heterogeneous group of disorders in the sizeable proportion of patients who exhibited a functional abnormality in the microcirculation when measured directly—with either inadequate vasodilator response to pharmacological or pacing stimuli or enhanced sensitivity to vasoconstrictive stimuli.^4^ The presence of CMD heralds a worse prognosis with an increased risk of major adverse cardiovascular events (MACE) in the longer term, but also represents a therapeutic target of unmet need.^5^ The severity of myocardial ischemia attributable to microvascular disease is independently associated with excess cardiovascular risk and correlates well with symptom burden.^6 7^ Cardiac Syndrome X is a historical term used to describe patients with angina, angiographically smooth coronary arteries and an exercise ECG demonstrating ischaemia. Older non-invasive stress testing (exercise ECG, stress echocardiography or nuclear imaging) had low sensitivity (41%) and specificity (57%) at identifying patients characterised by the invasive gold standard.^8^ Given that CMD may be associated with perfusion abnormalities confined to the subendocardial layer of myocardium, modalities with higher spatial resolution, such as cardiac MRI may allow more accurate detection of this condition.^9^

Conventional stress testing can overlook coronary vasospasm, a functional disorder of the coronary circulation, typically triggered by cold air or emotional stress rather than physical exertion (often resulting in a true ‘negative’ stress test) and is a distinct diagnosis to CMD. Physicians aware of the poor accuracy of non-invasive tests, often wrongly dismiss CMD as a cause of symptoms while conversely, if microvascular angina is presumed without verification, empirical treatment with angina medication may result in inappropriate treatment of patients with a non-cardiac problem. Both CMD and coronary vasospasm would benefit from clearer diagnostic pathways.

### Diagnosis and treatment in current practice

Traditional atherosclerotic risk factors and ‘typicality’ of angina are poor predictors of CMD, necessitating objective tests to establish a clinical diagnosis; however, tests of coronary microvascular function are rarely used in clinical practice.^10^ Patients are frustrated by a lack of clarity and effective management for this problem, often being subject to repeated invasive coronary angiography when symptoms persist, with associated health and economic burden.^11^ The European Society of Cardiology (ESC) guidelines suggest empirical use of traditional antianginal therapies for symptom relief in NOCAD along with aspirin, 3 -hydroxy-3-methylglutaryl-CoA reductase inhibitors (statins) and ACE inhibitors for secondary prevention.^12^ To date, very few randomised controlled trials have been performed in patients with confirmed CMD and these gaps in evidence are reflected in the relatively soft and contradictory recommendations provided by international guidelines.

The ability to physiologically characterise CAD at the time of angiography by pressure-wire assessment has revolutionised the interventional cardiology field.^13^ Advances in guidewire technology now also enable the immediate assessment of the coronary microcirculation to provide clarity into the NOCAD diagnosis. The Coronary Microvascular Angina randomised-controlled trial (CorMicA) recently demonstrated that a tiered approach for assessment of microvascular or vasospastic angina in patients with NOCAD is superior to usual care.^14^ Contemporary guidelines from the Coronary Vasomotion Disorders International Study (COVADIS) Group mandate invasive coronary reactivity testing to establish a definitive diagnosis of microvascular angina secondary to CMD or coronary vasospasm.^15^ CMD can be identified during a single cardiac catheterisation procedure, overcoming issues of specificity and sensitivity associated with non-invasive ischaemia tests. Coronary reactivity testing has a high diagnostic yield of identifying CMD and vasospasm (60%) with small additional procedural risks (up to 0.7%), carrying a IIb indication on ESC guidelines for investigating refractory angina.^12 16 17^ Other authors have proposed diagnostic algorithms to stratify NOCAD; however, our aim is to further simplify this message.^18^

In this review, we propose an approach that involves routine assessment of microvascular function in all patients shown to have NOCAD, aimed at establishing diagnoses in the majority and allowing standardised classification of phenotype. The latter will provide the foundation for collating data for research and international audit purposes and will ultimately act as the basis for stratified medicine in this large group of patients, who are poorly served by current clinical practice. We review the evidence underlying this proposal and finally provide a simple algorithm that could be implemented in most cardiovascular centres that manage such patients.

### The CMD and CAD continuum: insights from recent trials

**Figure 1:**
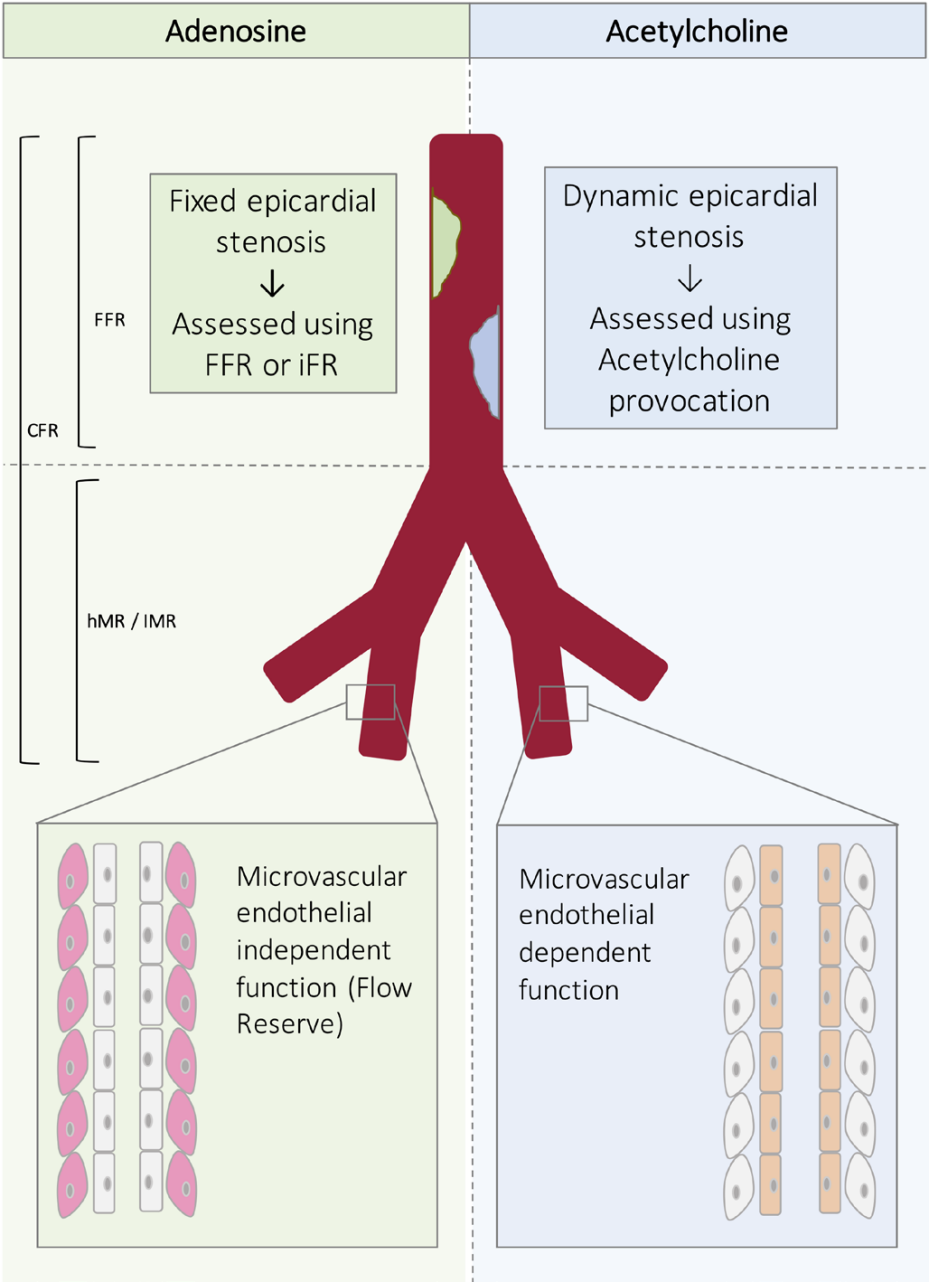
Compartmental model of the coronary epicardial and microcirculation and how to interrogate the respective components. CFR, coronary flow reserve; FFR, fractional flow reserve; hMR, hyperaemic microvascular resistance; IMR, index of microcirculatory resistance.

For simplicity, the coronary circulation is often described using a two-compartment model: the epicardial arteries and the microcirculation (figure 1). Both CMD and CAD often coexist and it is only for simplicity that they are considered as dichotomous entities when classifying patients in the clinical setting. Several recent randomised controlled trials of patients with CAD have encompassed patients with NOCAD, highlighting the relative frequency of this condition, demanding therapies distinct from CAD (table 1). A binary approach restricted to coronary angiography without appreciating the microcirculation may account for equivocal results demonstrated in some studies looking at prognostic and symptomatic benefits of percutaneous coronary intervention (PCI). There is growing interest in the comprehensive assessment of epicardial stenoses with emerging evidence that the microvascular component is a greater determinant of prognosis.^19^ This is currently being investigated in more detail by the randomised controlled trial DEFINE Flow, where only patients with both flow-limiting CAD (FFR≤0.80) and impaired vasodilator reserve (CFR<2.0) undergo PCI.

**Table 1:** Insights from recent randomised controlled trials specific to patients with NOCAD and CMD.

Trial, journal and year	Patients with NOCAD (n)	Pertinent results in patients with NOCAD	Take home messages for NOCAD
FAME-2, *NEJM*, 2012^13^	332/1220 (27%) of patients had an FFR >0.80	Similar CCS angina class compared with FFR <0.80. Similar levels of silent ischaemia compared with FFR <0.80. MACE rate of 9% in 2 years.	Patients with NOCAD have similar symptom burden to patients with CAD. Patients with NOCAD have a high MACE rate.
PROMISE, *NEJM*, 2015^36^	4477/4996 (90%) of patients in the CTCA group had a ‘negative’ result	89% of patients had typical or atypical angina in both groups. Only 10% of patients in the CTCA group had angina attributable to CAD.	A large proportion of anginal symptoms are attributable to NOCAD in patients with an intermediate PTP of CAD.
SCOT-HEART, *Lancet*, 2015^37^	1326/1778 (75%) in the CTCA groups had anatomical NOCAD	CTCA-guided therapy resulted in a reduced diagnosis of angina due to CAD, prompting alteration of therapy in this group, and deterioration in QoL and symptoms. Patients in the CTCA group with a change in diagnosis, either confirming obstructive CAD or excluding CAD had the greatest improvement in symptoms, while those with non-obstructive CAD had the least improvement in symptoms.	In a population with high predicted 10-year CHD risk, NOCAD is common. Defining NOCAD anatomically (instead of with FFR) may explain the poorer outcomes in the non-obstructive CAD group. Inappropriate cessation of antianginal therapy in patients with NOCAD with CMD may have attenuated symptom improvement derived by identifying CAD and revascularisation leading to symptom neutrality when adopting a CTCA-guided approach.
CE-MARC 2, *JAMA*, 2016 38	139/265 (52%) of patients who underwent angiography had NOCAD	All 1202 patients had angina with 401 (33%) having typical angina. A minority of patients had a positive non-invasive test (12.4% in the CMR group, 18.2% in the MPS group and 13.4% in the NICE guideline group).	Adhering to NICE guidelines results in a frequent diagnosis of NOCAD. The rate of ‘unnecessary angiography’ was nearly double in women, compared with the rate in men.
ORBITA, *Lancet*, 2017^39^	57/200 (29%) of patients had an FFR >0.80	In a sham-placebo trial, PCI was found to be equivalent to OMT in providing symptom relief. Patients with angina and FFR >0.80 also underwent PCI and may have diluted effects between the groups.	NOCAD is common among lesions judged to be visually severe. Differences in microvascular physiology is postulated to have contributed to inconsistent benefits of PCI.
ISCHAEMIA and CIAO-ISCHAEMIA substudy, tba^40^	5179 patients with moderate ischaemic burden have been randomised to an invasive approach versus OMT (ISCHAEMIA trial)	A proportion of patients with moderate-to-severe ischaemia on stress testing have NOCAD (enough to justify forming the CIAO-ISCHAEMIA substudy).	INOCA is an increasingly recognised entity that warrants specific management.

CAD, coronary artery disease; CCS, Canadian Cardiovascular Society; CFR, coronary flow reserve; CMR, cardiovascular magnetic resonance; CTCA, CT coronary angiography; FFR, fractional flow reserve; INOCA, ischaemia with no obstructive coronary disease; MACE, major adverse cardiovascular events; MPS, myocardial perfusion scintigraphy; NICE, National Institute for Health and Clinical Excellence; NOCAD, non-obstructive coronary artery disease; OMT, optimal medical therapy; PCI, percutaneous coronary intervention; PTP, pretest probability.

### Comprehensive assessment of NOCAD in the cardiac catheterisation laboratory

**Figure 2:**
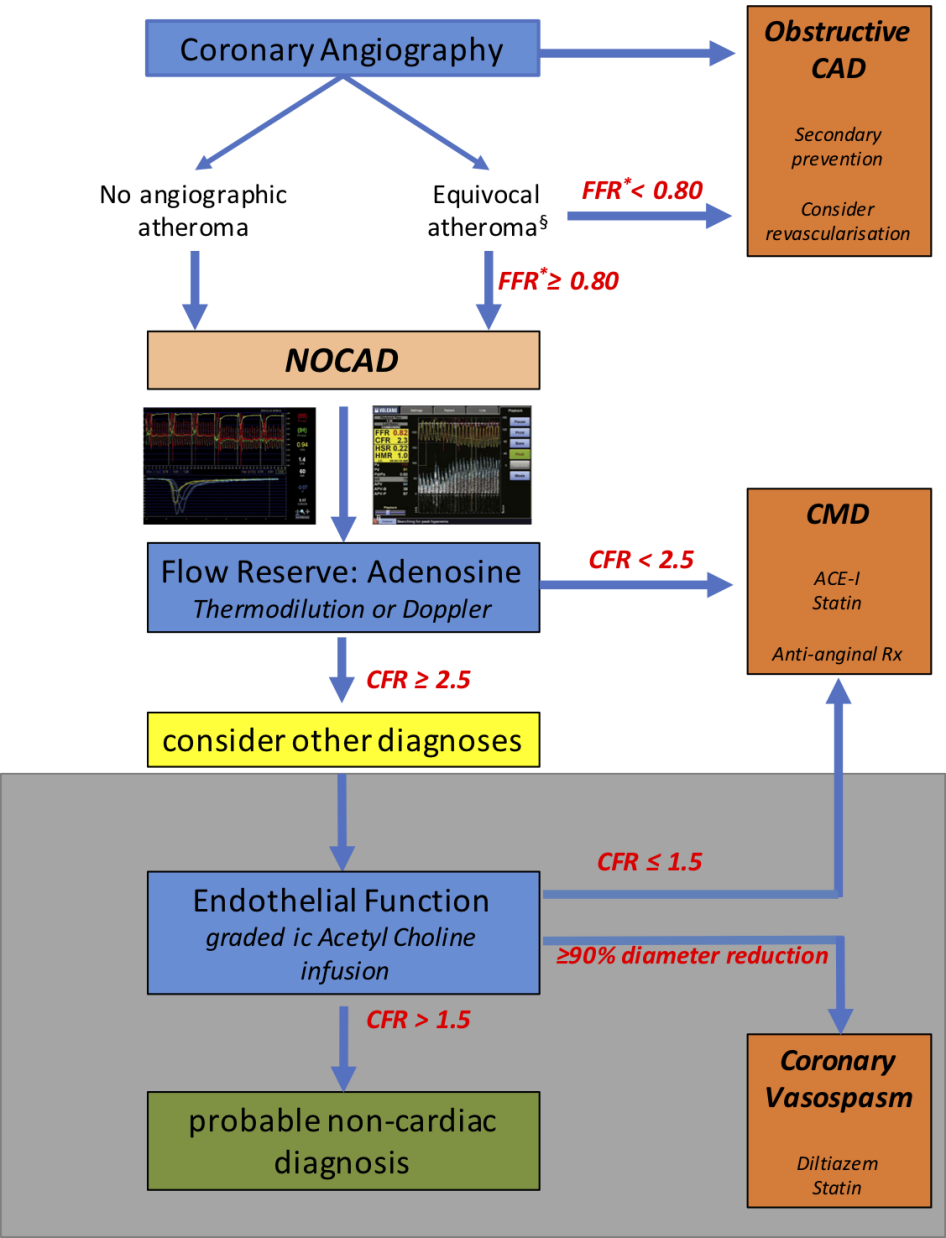
Comprehensive assessment of NOCAD during the time of angiography. *Evaluation of atheromatous disease can be carried out using resting or submaximal hyperaemic indices based on local practice. §Patients with visible atheroma should be commenced on secondary preventative therapy regardless of final diagnosis. The white area describes tests available on an ad hoc basis in all catheter laboratories, whereas the grey shaded area describes acetylcholine testing that can currently only be performed on a named-patient basis clinically, or within the context of dedicated research protocols, limiting its widespread ad hoc use. CAD, coronary artery disease; CFR,  coronary flow reserve; CMD,  coronary microvascular dysfunction;  FFR,  fractional flow reserve; NOCAD, non-obstructive coronary artery disease.

A patient presenting for coronary angiography can be characterised beyond a NOCAD diagnosis during the same procedure (figure 2).

#### Coronary angiography and physiological assessment to rule in/rule out obstructive CAD

Initial anatomical assessment of the coronary arteries may identify the presence of critical obstructive epicardial disease, or in the case of angiographically smooth unobstructed vessels, allow this to be definitively excluded. Physiological evaluation of CAD is superior to visual angiographic appraisal at detecting disease capable of causing ischaemia and using these techniques to guide revascularisation decisions has been shown to produce superior outcomes.^20^ The pressure-wire derived index of fractional flow reserve (FFR) has prognostic utility in the stable CAD population while also representing a cost-effective healthcare economical strategy.^21^ Resting and submaximal hyperaemic indices are emerging alternative options to FFR.^22^ Equivocal epicardial artery stenoses (diameter stenosis from 40% to 90%) should be interrogated in line with contemporary practice guidelines, all physiological techniques being superior to anatomical assessment alone. The presence of non-obstructive atherosclerotic disease heralds a poorer prognosis among NOCAD and should be managed with secondary preventative therapy.^11^ Plaque rupture of non-obstructive atheromatous disease can be a source of MACE in addition to the presence of ischaemia within intramyocardial vessels. The absence of flow-limiting epicardial disease, or NOCAD, should then prompt a stratified approach to identify an ischaemic substrate for the presenting symptoms.

#### Assessment of coronary flow reserve (endothelium-independent microvascular function)

In the absence of physiologically flow-limiting epicardial disease, reduced coronary flow reserve (CFR) indicates the presence of CMD. CFR is defined as the ratio of maximal blood flow during hyperaemia, to resting blood flow and is the clinical reference standard for quantitative assessment of microvascular vasodilatory reserve; in the absence of epicardial coronary disease, values below 2.0 are abnormal while values above 2.5 indicate normal microvascular function. Absolute coronary flow is difficult to measure in a clinical setting but Doppler-based techniques are used to measure coronary flow velocity, which is used as a surrogate of flow when calculating CFR, as it has been shown that there is negligible variation in epicardial vessel diameter in response to adenosine. Thermodilution techniques can also be used; as flow is inversely proportional to transit time of a cold bolus of saline, CFR can be defined as the ratio of mean transit time at baseline and hyperaemia. Given that CFR can only be used to quantify microvascular function in the absence of epicardial conduit artery disease, newer parameters have recently been evaluated to directly measure microvascular resistance (MR). MR is defined as the ratio between myocardial perfusion pressure (which approximates to distal coronary pressure (Pd)) and flow; when flow is estimated by Doppler flow velocity the resulting index is called hyperaemic microvascular resistance (hMR=Pd/APV, where APV is average peak velocity) and when flow is estimated by thermodilution, it is called index of microvascular resistance (IMR=Pd×Tm, where Tm is mean transit time). In the context of NOCAD, hMR >2.4 mm. Hg/cm.s or IMR >25 units suggests underlying microvascular dysfunction and correlates with symptom burden.^7 23^ Reduced CFR is often associated with atherosclerotic disease not immediately apparent on angiographic appearance, assessment of MR may delineate between diffuse epicardial and microvascular pathology.^24^ Patients with a normal CFR but increased hMR/IMR represent a distinct group of patients who may also benefit from therapy for CMD.^14^

FFR, CFR and MR can now be calculated using a single 0.014-inch coronary wire, either a dual pressure and Doppler sensor-tipped guidewire (ComboWire Guidewire; Philips Volcano, San Diego, California, USA) or a pressure wire with temperature thermistor on the distal shaft and tip (Abbot, Santa Clara, California, USA). While Doppler has the temporal resolution to identify changes in coronary blood flow during the cardiac cycle, use is limited by operator expertise and a steep learning curve. Thermodilution has demonstrable comparability to Doppler but is not identical in all scenarios.^25^ Where adenosine-mediated (endothelial-independent) vasodilatation is unimpaired, the operator should consider testing for endothelium-dependent microvascular dysfunction function.

#### Assessment of endothelium-dependant microvascular function

Endothelial dysfunction precedes atherosclerosis and often coexists with abnormalities in microvascular smooth muscle dysfunction. In coronary arteries with normal endothelium, intracoronary acetylcholine (ACh) dilates the epicardial and microvascular circulation, increasing CBF. In the presence of healthy endothelium, ACh causes vasodilation by generating nitric oxide that acts on the surrounding vascular smooth muscle. Depending on the integrity of the endothelium and reactivity of the surrounding smooth, ACh can result in either vasodilatation or vasoconstriction. ACh is administered as a slow infusion over 2 min, via the coronary guiding catheter (to prevent systemic effects), starting with an initial low dose of 0.18 µg/min followed by a high dose at 18 µg/min.^16^ A below 50% increase in CBF from baseline or ischaemic ECG changes and pain in the absence of epicardial vasoconstriction (>90% diameter reduction) is diagnostic of microvascular endothelial dysfunction and is associated with a poorer prognosis.^26^ If no abnormalities are detected using these doses of ACh, higher ‘provocation’ doses can be used to unmask a diagnosis of coronary vasospasm.

#### Provocation testing for coronary vasospasm

The reference method for provocative spasm testing involves intracoronary administration of a provocative stimulus during invasive coronary angiography with the monitoring of patient symptoms, ECG and angiographic documentation of coronary artery spasm. A positive provocation test as defined by COVADIS entails: (1) reproduction of the usual chest pain, (2) ischaemic ECG changes and (3) >90% vasoconstriction on quantitative coronary angiography.^27^ Microvascular spasm is diagnosed where chest pain and ischaemic ECG changes occur in the absence of epicardial artery constriction.^17^ High dose ACh provocation is commonly used to induce abnormal coronary vasospasm, where incremental bolus doses of 20, 50 and 100 µg are injected into the left coronary artery at 5 min intervals. If negative, this is repeated in the right coronary artery in a similar manner, at doses of 20 and 50 µg. Liberal intracoronary glyceryl trinitrate usually alleviates ACh-induced vasospasm.

### Non-invasive diagnosis of CMD

The wider adoption of anatomical imaging with CT coronary angiography (CTCA) as a first-line diagnostic test for the assessment of stable chest pain may increase the diagnosis of NOCAD. The higher sensitivity and spatial resolution afforded by perfusion cardiac MRI can be used to rule-out those unlikely to have CMD. Novel gadolinium-free mapping techniques may have a role upstream of anatomical imaging assessment, of distinguishing CAD, CMD and non-ischaemic chest pain and will need to be studied further.^28^ While these hybrid and novel imaging approaches greatly improve the heterogeneity conferred by a NOCAD diagnosis by assessing for CMD, this approach is currently unable to identify coronary vasospasm.

### Therapeutic interventions in CMD

Robust clinical trial evidence for the treatment of CMD is lacking and the optimal management strategies in these patients remains undefined. The evidence base largely comes from small diagnostic studies, which have sometimes yielded conflicting results and have often used variable inclusion criteria, differing diagnostic test thresholds and dissimilar endpoints. Most therapeutic trials have involved patient cohorts without a precise diagnosis of CMD, but instead have been classified as Syndrome X or INOCA (table 2). Therapeutic agents in CMD may be considered symptom-modifying or disease-modifying agents. In current practice, antianginal therapy and secondary prevention tends to be empirical and follows a similar paradigm to the evidence-based therapies for obstructive epicardial CAD. Non-pharmacological therapies such as exercise training in cardiac rehabilitation programmes, improve resting diastolic blood pressure and exercise capacity in patients with CMD, while cognitive behavioural therapy and spinal cord stimulation may help in subsets of patients with high autonomic tone or abnormal nociception, respectively.^29^ The need for further therapeutic options is highlighted by the approximately one-third of patients with NOCAD who have refractory angina.^30^

**Table 2:** Therapeutic studies in patients with syndrome X or INOCA and those with confirmed CMD

Drug class	Syndrome X/INOCA population	CMD population
First-line antianginal agents		
Beta blocker	Randomised double-blind crossover studies—reduced angina, less ST depression episodes, improved markers of endothelial function 41	Not tested in this population
Calcium channel blockers	Reduced angina, increased exercise time 42	Only single dose of intravenous diltiazem tested did not improve CFR immediately^43^
Nitrates	Reduced ischaemic threshold to exercise or rapid pacing 44	Not tested in this population
Second-line antianginal agents		
Nicorandil	Not tested	Increased ischaemic threshold (using CFR <3.0 as CMD inclusion) 45
Ranolazine	Contradictory, improved or unchanged symptoms^46^	Improved symptoms and reduced coronary microvascular resistance measured invasively (using CFR<2.5 or IMR>20 U as CMD inclusion) 47
Disease-modifying agents		
ACE inhibitors	Increased exercise duration, ischaemic threshold, endothelial function and CFR 48	Improved CFR at 16 weeks (using CFR<3.0 as CMD inclusion) 49
Statins	Improved symptoms, exercise tolerance and endothelial function 50	Improved coronary ACh CFR after 6 months treatment (using ACh CFR <1.5 as CMD inclusion) 32

Green text emphasises improvement with medication and red text emphasises deterioration with medication.

CFR, coronary flow reserve; CMD, coronary microvascular dysfunction; INOCA, ischaemia with no obstructive coronary arteries.

### Disease-modifying agents in CMD

The current ESC guidelines recommend treatment with aspirin and statin (class I indication), and consideration of ACE inhibitors (IIb indication) for NOCAD.^12^ Intracoronary ultrasound of patients with NOCAD suggest a high prevalence of epicardial atherosclerosis; aspirin is recommended by extrapolation of CAD studies.^31^ There is evidence from small randomised trials that treatment with ACE inhibitors and statins may be beneficial in CMD.^32–35^ Statin therapy reduces cardiovascular risk via low-density lipoprotein reduction but has several pleiotropic effects including improvements in vascular inflammation and enhanced endothelial function. Angiotensin II is a potent vasoconstrictor and may modulate coronary microvascular tone directly and indirectly via left ventricular effects on cardiac–coronary coupling.

### Antianginal therapies for CMD

While the evidence base for antianginal therapy in CMD is less mature than for other conditions, notably obstructive CAD, we believe there is sufficient evidence, as outlined in contemporary guidelines, to make informed, personalised decisions for patients with ongoing symptoms and demonstrable ischaemia. Non-dihydropyridine calcium channel blockers are efficacious in both CMD and coronary vasospasm and therefore should be used first line for CMD where coronary vasospasm has not been ruled out. Beta-blockers can be beneficial in CMD but may potentiate coronary vasospasm due to unopposed α-receptor agonism, meaning they should be used as first-line therapy for CMD provided coronary vasospasm has been ruled out by vasoreactivity testing with acetylcholine. Nicorandil and ranolazine should be used as second-line and third-line therapies for CMD if symptoms persist.^12 14^

### Therapies for coronary vasospasm

The clinical manifestations of coronary vasospasm include sudden cardiac death, myocardial infarction and syncope, with therapies such as calcium channel blockers and statins shown to improve this adverse prognosis.^33^ Calcium channel blocker are highly efficacious for treating coronary vasospasm with long-acting nitrates being suitable second-line agents, while statins augment the effect of both and should also be considered a first-line agent.^34^ Novel therapeutics are being investigated for coronary vasospasm and these studies give an indication of the paradigm shift in therapeutics that needs to be considered for patients with NOCAD. Endothelin is a potent vasoconstrictor and abnormalities in the endothelin pathway are associated with coronary microvascular dysfunction.^35^ Endothelin receptor antagonist therapy has been associated with enhanced coronary endothelial function in patients with angiographically unobstructed coronaries and this may represent a targeted therapeutic agent for patients with epicardial and microvascular endothelial dysfunction.

### Future research in NOCAD and CMD

Future clinical trial designs of existing and novel therapeutic agents in patients with NOCAD should enrol patients with symptoms and defined phenotypes. The stratified medicine approach is aimed at identifying specific disease phenotypes within a heterogeneous population with linked therapy; better diagnosis will yield better disease-specific treatments that will improve the outcomes of all patients with NOCAD. CorMicA has demonstrated that ad hoc use of coronary function tests in appropriately selected patients during routine clinical practice leads to health and economic benefits.^14^ While vasodilators are central to diagnosing CMD in clinical practice, patients develop symptoms during physical exercise and mental stress, physiologically distinct states to pharmacological hyperaemia. Mechanistic studies directly measuring Doppler–derived CBF and cardiac–coronary coupling, look to unravel whether patients with CMD display maladaptation during physical exercise. Larger registries combined with enhanced understanding of the underlying pathophysiology will provide insight into CMD subtypes and in the future may yield better targeted therapy. Ultimately, large adequately powered health outcomes trials are needed to determine whether hard morbidity and mortality endpoints may be improved by targeted therapy.

## Conclusion

Contemporary practice dictates that diagnostic clarity is needed beyond an all-encompassing NOCAD diagnosis to enable targeted therapy and personalised medicine. Clinically available guidewires provide an opportunity to better identify the ischaemic substrate at the time of angiography, by measurement of FFR and CFR that could influence the clinical management of a subset of patients with NOCAD. The superior classification of NOCAD phenotypes will also facilitate mechanistic trials to yield more informative results compared with those that previously recruited heterogonous patient groups and could serve as a platform for much needed future therapies for patients with INOCA. The ultimate goal would be to improve our management of this increasingly recognised and poorly managed condition, while improving resource allocation during a time of healthcare austerity.
